# Antioxidant and anti hyperglycemic role of wine grape powder in rats fed with a high fructose diet

**DOI:** 10.1186/s40659-015-0045-4

**Published:** 2015-09-30

**Authors:** Romina Hernández-Salinas, Valerie Decap, Alberto Leguina, Patricio Cáceres, Druso Perez, Ines Urquiaga, Rodrigo Iturriaga, Victoria Velarde

**Affiliations:** Departamento de Fisiología, Facultad de Ciencias Biológicas, Pontificia Universidad Católica de Chile, Santiago, Chile; Center for Molecular Nutrition and Chronic Diseases, Pontificia Universidad Católica de Chile, Santiago, Chile

**Keywords:** Insulin resistance, Oxidative stress, Metabolic syndrome, Wine grape powder

## Abstract

**Background:**

Metabolic syndrome is a growing worldwide health problem. We evaluated the effects of wine grape powder (WGP), rich in antioxidants and fiber, in a rat model of metabolic syndrome induced by a high fructose diet. We tested whether WGP supplementation may prevent glucose intolerance and decrease oxidative stress in rats fed with a high fructose diet.

**Methods:**

Male Sprague–Dawley rats weighing 180 g were divided into four groups according to their feeding protocols. Rats were fed with control diet (C), control plus 20 % WGP (C + WGP), 50 % high fructose (HF) or 50 % fructose plus 20 % WGP (HF + WGP) for 16 weeks. Blood glucose, insulin and triglycerides, weight, and arterial blood pressure were measured. Homeostasis model assessment (HOMA) index was calculated using insulin and glucose values. A glucose tolerance test was performed 2 days before the end of the experiment. As an index of oxidative stress, thiobarbituric acid reactive substances (TBARS) level was measured in plasma and kidney, and superoxide dismutase was measured in the kidney.

**Results:**

Thiobarbituric acid reactive substances in plasma and renal tissue were significantly higher when compared to the control group. In addition, the area under the curve of the glucose tolerance test was higher in HF fed animals. Furthermore, fasting blood glucose, plasma insulin levels, and the HOMA index, were also increased. WGP supplementation prevented these alterations in rats fed with the HF diet. We did not find any significant difference in body weight or systolic blood pressure in any of the groups.

**Conclusions:**

Our results show that WGP supplementation prevented hyperglycemia, insulin resistance and reduced oxidative stress in rats fed with HF diet. We propose that WGP may be used as a supplement in human food as well.

## Background

The development of industrialized countries together with technological advances has produced strong social changes that directly affect the human lifestyle. Changes in diet and a sedentary lifestyle characterized by the lack of exercise in daily life advance in parallel with a growing number of metabolic diseases, resulting in high cardiovascular risk and increased morbidity and mortality rate. The association of a collection of risk factors including hypertension, high triglycerides, low HDL cholesterol, and high fasting glucose, which are linked to type 2 diabetes, cardiovascular disease and stroke, created a condition called metabolic syndrome (MS). The World Health Organization defines MS as a prothrombotic state, with dyslipidemia, alteration in the metabolism of glucose, a sustained rise in blood pressure, abdominal obesity and a systemic pro-inflammatory state [1]. The metabolic syndrome has high prevalence, estimated between 20 and 30 % of adult worldwide population [2, 3]. Abdominal obesity, one of the MS features, produces oxidative stress. Accordingly, it has been found that an increased oxidative stress in abdominal adipocytes may increase various pro-inflammatory cytokines and fatty acids, which exacerbate other MS factors such as insulin resistance, hypertriglyceridemia, and hypertension [4]. In addition, it has been demonstrated that the activity of several antioxidant enzymes decreases in humans with MS [5]. One of the transcription factors responsible for the initiation of the antioxidant response is the nuclear factor E2-related factor-2 (Nrf2), which controls the expression of cellular phase-2 detoxifying and antioxidant enzymes [6, 7]. Nrf2 induces the expression of several antioxidant enzymes [8–11] such as superoxide dismutase (SOD) to restore the imbalance of oxidative stress [12, 13].

Because MS involves several different factors, no unique pharmaceutical treatment exists to prevent or treat it. The best solution seems to be to adjust lifestyle factors like diet and exercise. It is known that grape antioxidants play a role in the reduction of the metabolic syndrome risk factors. Grapes contain numerous antioxidants such as polyphenols, anthocyanins, flavonols and resveratrol. A simple way to make grape polyphenols part of the diet is to consume grape products. Wine grape pomace is an underused residue of the wine making process. This grape by-product contains pressed skins, seeds and stems and accounts for about 20 % of the weight of the grapes used to make wine. In addition, grape pomace is a rich source of polyphenols including monomeric and oligomeric proanthocyanidins and a diversity of anthocyanin glycosides [14]. Due to the abundance of antioxidants found in this by-product, grape pomace is increasingly being used to obtain functional food ingredients and as a dietary supplement [15]. Flavonoids that are present in grape pomace are a large group of polyphenolic antioxidants present in a variety of foods. Most naturally occurring flavonoids can induce chelation of transition metals, the scavenging of free radicals, and inhibition of radical producing enzymes [16]. In addition, it has been postulated that a flavonoid-rich diet can prevent several degenerative age-related diseases [17] and several kinds of cancer [18]. It can also reduce the incidence of coronary heart disease [15], and chronic renal disease [19]. As mentioned before, it is well accepted that diets rich in polyphenols have health benefits, but the mechanisms of action of these molecules in the human body are not fully understood [20–22]. Wine grape powder (WGP) made from dried wine grape pomace, is rich in fiber and polyphenols. However, little is known about the potential effects of WGP in the oxidative state or metabolic markers of MS. Therefore, the present study was conducted to investigate the antioxidant and anti-insulin-resistance effect of WGP in an established rat model of MS induced by high fructose diet [23–26].

## Methods

### Wine grape powder

Wine grape pomace, a waste product from wine production was obtained from a single production lot of red grapes (Cabernet Sauvignon, vintage 2011, Maipo Valley, Chile) from Concha y Toro Winery, and stored at −20 °C until used, for approximately 3 months. The frozen wine grape pomace was thawed at room temperature and dried in a forced air dryer at 60 °C until moisture reached less than 12 %. The dried pomace was grinded with a hammer mill to obtain a wine grape pomace powder (WGP), which was packaged in double plastic bags of 20 kg. WGP has a high content of dietary fiber (48 %) determined by AOAC 991.43. The content of polyphenolic compounds was 41.11 ± 3.01 mg galic acid equivalent/g, determined by the Folin-Ciocalteu procedure [27]. WGP with a high antioxidant capacity of 362.9 ± 24.4 µmol TE/g, measured by the oxygen radical absorbance capacity assay [28].

### Rats, diets, and treatment with WGP

The experimental protocols were approved by the Bio-ethical Committee of the Faculty of Biological Sciences of the Pontificia Universidad Católica de Chile and were in accordance with the Guide for the Care and Use of Laboratory Animals published by the US National Institutes of Health (NIH Publication No. 85-23, revised 1996). Male Sprague–Dawley rats (6 week old, 180 g) were obtained from the Faculty of Biological Sciences Animal Care Facility. Rats were randomly divided into 4 groups according to their feeding treatments: control diet-fed rats (C; n = 8), control diet-fed rats supplemented with WGP (C + WGP; 20 % of WGP; n = 8); high-fructose diet-fed rats (HF; 50 % fructose, n = 7) and high-fructose diet-fed rats supplemented with WGP (HF + WGP, n = 6).

Mixtures for each of the diets were prepared on an industrial mixer at the Molino La Estampa, Chile, using the proportions for each of the ingredients shown in Table 1. Food pellets for each of the animal diets were prepared daily by adding the same amount of water to a fraction of each of the powder mixtures.

**Table 1:** Composition and energy provide by the experimental diets

Compound	C	C + WGP	HF	HF + WGP
Corn flour g/100 g Molino La Estampa, Chile	62.1	50.0	12.1	0.0
Fructose g/100 g 105321 Merck	0.0	0.0	50.0	50.0
Caseinate g/100 g C8659 Sigma-Aldrich	20.0	18.0	20.0	18.0
Methionine g/100 g M9625 Sigma-Aldrich	0.3	0.3	0.3	0.3
Corn oil g/100 g Chef, Chile	5.0	5.0	5.0	5.0
Cellulose g/100 g Molino La Estampa, Chile	8.1	2.2	8.1	2.2
WGP g/100 g	0.0	20.0	0.0	20.0
Mineral mixture g/100 g AIN-76 MP biomedicals	3.5	3.5	3.5	3.5
Vitamin mixture g/100 g AIN-76 MP biomedicals	1.0	1.0	1.0	1.0
Energy from fat %	11.2	15.0	11.3	15.0
Energy from carbohydrates %	62.2	63.9	68.4	63.9
Energy from proteins %	20.3	21.1	20.3	21.1
Energy kcal/100 g	399.3	389.6	399.3	389.3

*C* control diet, *C* *+* *WGP* control diet plus 20 % wine grape powder, *HF* high fructose diet, *HF* *+* *WGP* high fructose diet plus 20 % wine grape powder

The proximate analysis of the WGP is shown in Table 2, and the composition and energy provided by proteins, carbohydrates and fat in experimental diets is shown in Table 1. Animals were fed with 36 g (dry mixture) of their respective diets daily for 16 weeks. Rats were individually housed under controlled temperature, 12-h-light/dark cycles, consumed water ad libitum*, and were* under veterinarian supervision.

**Table 2:** Proximate analysis of wine grape flour

	g/100 g
Fat	7.8
Protein	11.7
Carbohydrates^a^	17.0
Total dietary fiber	47.7
Soluble dietary fiber	3.5
Insoluble dietary fiber	44.2
Ash	8.4
Moisture	7.5
Energy kcal/100 g	321.8

Total dietary fiber corresponds to the sum of soluble and insoluble fractions

^a^Nitrogen free extract minus dietary fiber

### Physiological and metabolic variables

All rats were continuously monitored for body weight, food and water intake. Blood glucose and triglycerides were measured using a commercial enzymatic kit and strips (Code Free, SD Biosensor Inc, Korea). Oral glucose tolerance test (GTT) was performed 2 days before the end of the experiment, on rats that have been food-deprived for 12 h. Each animal received 2 g/kg of glucose diluted in 2 mL of water by gavage. Rats were anesthetized with isofluorane 2 % in O_2_, and blood samples were obtained from the tail vein at 0 min (before glucose administration) 30, 60 and 120 min. A glucose tolerance curve was obtained and the trapezoidal rule was used to determine the area under the curve (AUC).

### Arterial blood pressure measurements

Measurements of systolic arterial blood pressure was performed with a non-invasive method in conscious rats, in a room at 25 °C, with noise and light control. These measurements were done using a Power Lab coupled to a NIBP system with pulse transducer/cuff for rat by tail cuff (ADInstruments, Australia). Rats were trained at least for 1 month before the experimental measurements. In separate experimental series, three rats were anesthetized with isofluorane 2 % in O_2_ and were implanted with radio-telemetric transmitters (TA11PA, DSI, USA) in one femoral artery. After surgery, rats received Ketoprofen 0.2 mg/kg (Rhodia Merieux) and Enrofloxacin 20 mg/kg (Bayer) i.m. Rats were allowed to recover for 7 days prior to the surgery and then were fed with control, high fructose and high fructose + WGP diets for 120 days. Arterial blood pressure was averaged from 30 min recordings, performed between 9 and 10 AM every 3 days in the initial 90 days, and then every 10 days during the last 30 days.

### Blood and tissue samples

At the end of the experiment, blood samples were taken, and then animals were sacrificed under anesthesia (Xylasine/Ketamine, 10 mg/90 mg per kg). Kidneys were immediately removed, frozen in liquid nitrogen and stored at −80 °C for later determinations. Plasma insulin level was determined using a rat insulin ELISA kit (EMD Millipore Corporation, USA), calculating the homeostasis model assessment of insulin resistance (HOMA-IR), based on the following formula:

$${\text{HOMA }}{-}{\text{ IR }} = {\text{serum glucose }}\left( {{\text{mg}}/{\text{dL}}} \right) \, \times {\text{ plasma insulin }}( \upmu {\text{U}}/{\text{mL}})/ 40 5.$$

### Oxidative stress measurement

Levels of thiobarbituric acid reactive substances (TBARS) in the plasma and kidneys were estimated using a previously described method [29] with slight modifications. 100 μL of plasma or supernatant from tissue homogenate were mixed with 50 μL sodium dodecyl sulphate (8 %w/v), 375 μL thiobarbituric acid (0.8 % w/v), and 375 μL acetic acid (20 %v/v); and then heated for 60 min at 90 °C. The precipitated material was removed by centrifugation, and the absorbance of the supernatant was determined at 532 nm. Levels of TBARS were expressed as malondialdehyde (MDA).

### Electrophoresis and Western blot analysis

To determine relative levels of Nrf-2 (1:500; Santa Cruz Biotechnology) and mSOD (1:2000, Millipore), kidney homogenates were lysed in RIPA with protease inhibitors (1 mg/mL aminocaproic acid, 1 mg/mL benzamidine, 0.2 mg/mL SBTI and 3 mmol/L PMSF) and phosphatase inhibitors (12 μg/mL sodium orthovanadate, 4.46 mg/mL sodium pyrophosphate, and 4.2 mg/mL sodium fluoride). Lysates were centrifuged and supernatants were collected for Western blot analysis. Protein concentrations were determined by the method of Lowry [30]. Protein samples (100 μg) from homogenates under different treatments were separated by electrophoresis in 10 % SDS–polyacrylamide gel (SDS-PAGE). Proteins were transferred to a 0.45 μm PVDF membrane, which was blocked at room temperature with Tris pH 7.6/5 % skim milk (w/v)/BSA 1 % (w/v). Then, the PVDF membrane was incubated overnight at 4 °C with primary antibody, followed by incubation with rabbit secondary antibody conjugated to peroxidase (1:2000) for 2 h. Immunoreactive bands were visualized using a chemiluminescent reagent (Western Lightning, Perkin Elmer) according to the procedure described by the supplier and Kodak films X-LS. The bands detected were digitized and subjected to densitometric analysis using the program Image J (NIH, Washington DC, USA).

### Statistical analysis

Data obtained from different groups of rats were compared with each other by one-way analysis of variance (ANOVA) followed by a Bonferroni’s post hoc test. The analyses were performed with GraphPad Prism 5 software for Windows (GraphPad Software). Results are expressed as mean ± standard error of the mean (SEM). Differences were considered significant if p ≤ 0.05.

## Results

### Effects of the high fructose diet on the physiological parameters measured in rats

**Fig. 1:**
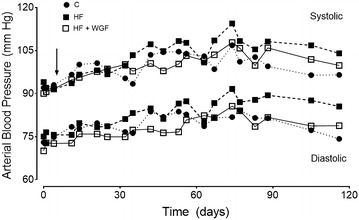
Arterial blood pressure measured by radiotelemetry in three conscious rats fed with control (C *filled circle*), high fructose (HF *filled square*) and high fructose + WGP (HF + WGP *unfilled square*) diets. Systolic and diastolic arterial blood pressure of the rats was displayed in the *upper* and *lower* part of the figure, respectively. Telemeters were implanted into the femoral artery and the recordings started 7 days after the surgery. *Arrow* indicate the beginning of the diet treatments

High fructose diet increased glucose, triglycerides and insulin levels in plasma measured after 12 h of food deprivation, but did not induce changes in systolic arterial pressure, body or heart weight (Table 3). Supplementation with WGP to the HF diet reduced the increase in glucose, triglycerides and insulin levels in plasma. In a separate experimental series, we recorded systolic and diastolic arterial pressure with radiotelemetry in three rats fed with control, high fructose and high fructose plus WGP. Arterial blood pressure slightly increased during the experiment in all groups. However a similar trend in the increase of arterial blood pressure was observed in all three rats (Fig. 1).

**Table 3:** Physiological parameters of rats fed experimental diets

	C	C + WGP	HF	HF + WGP
Glucose in plasma (mg/dL)	94.8 ± 1.8	88.3 ± 3.0	104.3 ± 2.8*	91. ± 2.84
Triglycerides in plasma (mg/mL)	92.4 ± 6.8	108.8 ± 4.2	140.7 ± 11.2*	122.1 ± 7.8
Insulin in plasma (μU/mL)	7.6 ± 1.5	5.8 ± 0.6	912.2 ± 1.3*	8.9 ± 0.7
Systolic arterial pressure (mmHg)	108.8 ± 4.4	110.6 ± 2.1	115.0 ± 1.7	112.2 ± 1.8
Body weight (g)	515.8 ± 22.6	519.1 ± 17.9	514.0 ± 22.4	528.9 ± 21.8
Heart weight (g)	1.43 ± 0.05	1.46 ± 0.06	1.50 ± 0.05	1.56 ± 0.09

n = 6–8 in each group

*C* control diet, *C* *+* *WGP* control diet plus wine grape powder, *HF* high fructose diet, *HF* *+* *WGP* high fructose diet plus wine grape powder

* P < 0.05 Bonferroni after one way ANOVA

### WGP prevented the rise of glucose and the area under the curve on the glucose tolerance test in fructose-fed rats

**Fig. 2:**
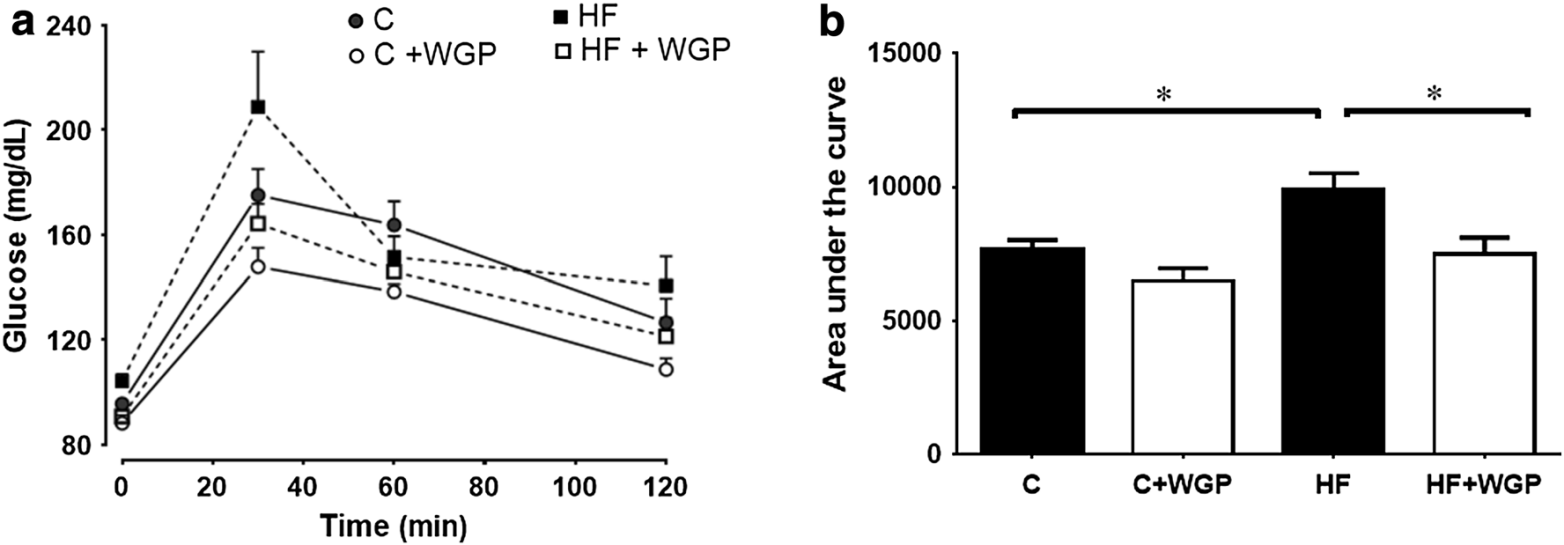
WGP prevents the increase in the area under the curve for the glucose tolerance test. **a** Glucose tolerance test curves are shown for control (C *filled circle*), control + WGP (C + WGP *unfilled circle*) high fructose (HF *filled square*) and high fructose + WGP (HF + WGP *unfilled square*) fed animals. Each *curve* represents mean ± SEM for at least six animals. **b** Area under the curve was calculated for the curve of each animal. *Bars* represent mean ± SEM for n = 6–8 rats in each group. *P < 0.05 HF vs. other groups. Bonferroni after one way ANOVA

Rats treated with a high fructose diet had the highest fasting level of blood glucose, before beginning the GTT (Fig. 2a). The GTT showed that the largest increase in blood glucose measured at 30 min and 2 h following glucose administration was observed in the HF group. In addition, it is worth to note that glucose levels in the HF + WGP group were slightly lower than the one on the control group, while the lowest curve was from the C + WGP group. To quantify these differences, we measured the area under curve (AUC) of the glucose tolerance test (Fig. 2b). We found a significant increase in the area under the curve in the HF fed group, whereas WGP prevented the increased AUC in the WGP + HF treated rats.

### WGP prevented the induction of insulin resistance in fructose-fed rats

**Fig. 3:**
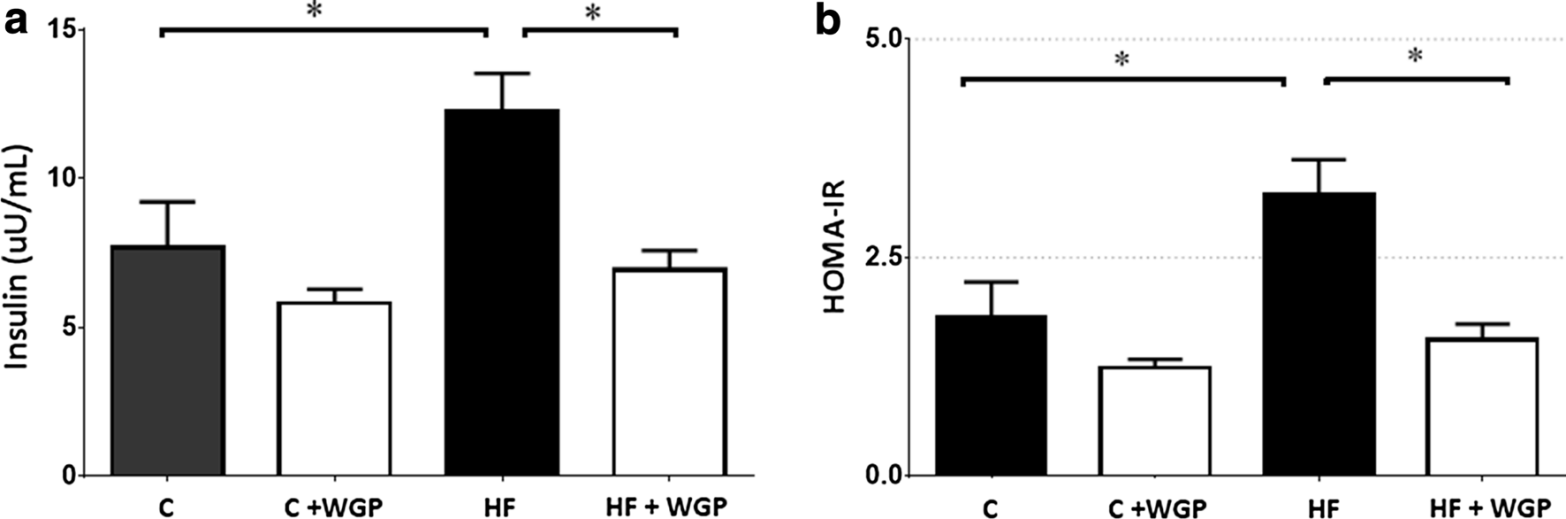
WGP prevents insulin resistance in HF fed rats. **a** Insulin was measured using a radioimmunoassay in control (C), control + WGP (C + WGP) high fructose (HF) and high fructose + WGP (HF + WGP) fed animals. **b** HOMA was calculated from insulin values shown in this figure and glucose values obtained at the same time points. *Bars* represent mean ± SEM for n = 6–8 rats in each group. *P < 0.05 HF vs. other groups. Bonferroni after one way ANOVA

To evaluate whether the increase in the AUC correlates with the insulin concentration, we measured insulin in the blood obtained after 12 h of starvation. Under these conditions, we found that rats from the HF fed group presented the highest levels of insulin, and its increase was prevented by the supplementation with WGP in the diet, as observed from the HF + WGP fed group (Fig. 3a). In addition, glucose levels were measured in these same blood samples and the homeostasis model assessment-estimated IR (HOMA-IR) index was evaluated for each group. HF fed rats presented a significantly higher HOMA-IR index compared to control rats. In addition, this increase was prevented in HF + WGP fed rats (Fig. 3b).

### WGP prevented the rise of TBARS in plasma from fructose-fed rats

**Fig. 4:**
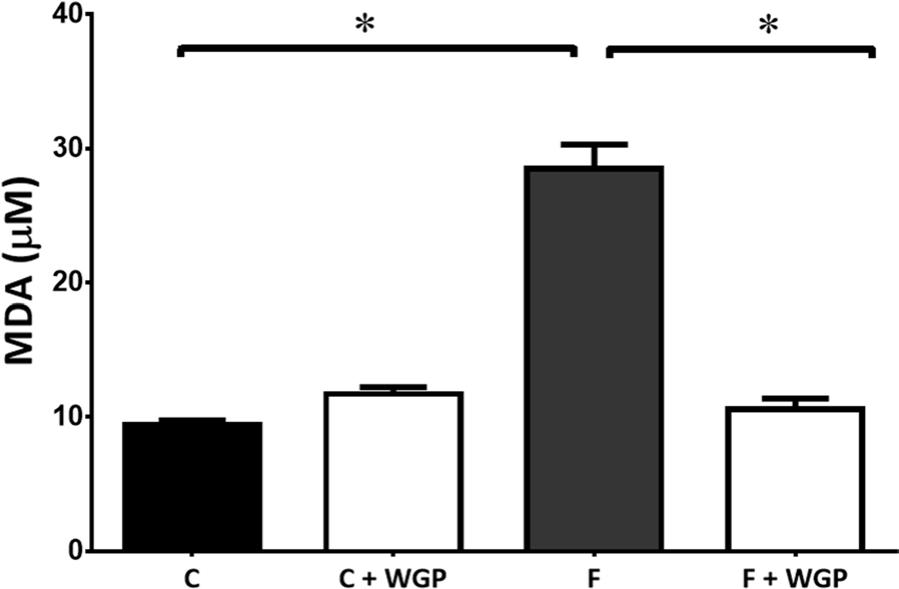
WGP reduces oxidative stress in HF fed rats. TBARS were measured in plasma from control (C), control + WGP (C + WGP) high fructose (HF) and high fructose + WGP (HF + WGP) diets. *Bars* represent mean ± SEM for n = 6–8 rats in each group. *P < 0.05 HF vs. other groups. Bonferroni after one way ANOVA

High fructose diet produced a significant threefold increase in plasma TBARS levels measured at the end of 16 weeks of treatment (Fig. 4). WGP effectively prevented this increase in TBARS levels in high fructose-fed rats. When compared to control, TBARS levels in HF + WGP were not significantly different from those of control rats.

### WGP prevented the reduction of SOD protein levels in kidneys from fructose-fed rats

**Fig. 5:**
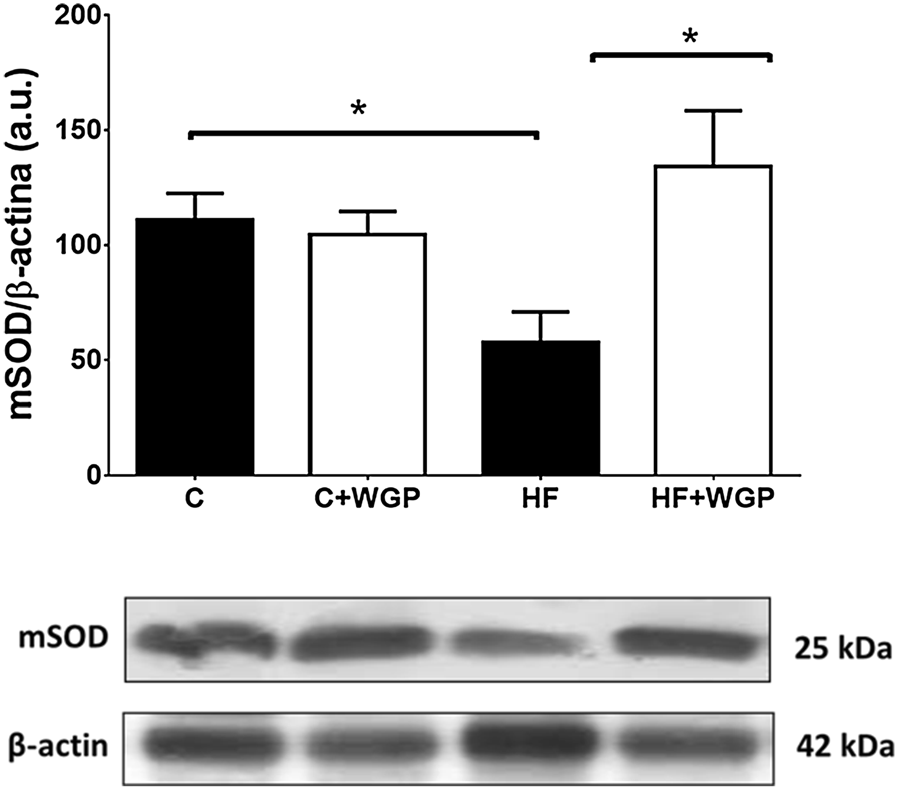
WGP prevents the reduction in renal mSOD protein levels. Protein extracts from kidneys from control (C), control + WGP (C + WGP) high fructose (HF) and high fructose + WGP (HF + WGP) fed animals were used to evaluate mSOD protein levels. *Bars* represent mean ± SEM for n = 4 animals in each group. *Bars* represent mean ± SEM for n = 4, *p < 0.05 HF vs. other groups. Bonferroni after one way ANOVA. Representative blots for mSOD and β-actin used as loading control are shown under the graph

Nrf-2 is considered a key transcription factor in the cellular response to oxidative stress [31]. This transcription factor regulates the expression of several antioxidant enzymes that participate in the defense against reactive oxygen species such as superoxide dismutase (SOD). Therefore, we evaluated the effect of HF treatment on Nrf-2 levels in renal tissue and found no significant changes after 16 weeks of treatment in this transcription factor. Although Nrf-2 protein levels were not significantly modified by the diet, we speculated that its activity could be modified. For this reason, we measured mSOD protein concentration, one of the proteins regulated by Nrf-2. We found a significant reduction in renal mSOD levels. Consistent with the previous observations, this decrease was not observed in HF rats fed with WGP (Fig. 5).

### WGP prevents the rise of TBARS in kidneys from fructose-fed rats

**Fig. 6:**
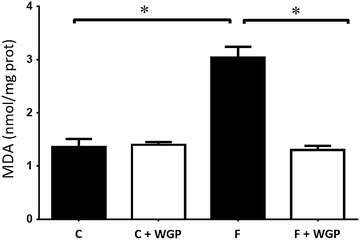
WGP prevented oxidative stress in renal tissue. Extracts from kidneys from control (C), control + WGP (C + WGP) high fructose (HF) and high fructose + WGP (HF + WGP) fed animals were used to determine TBARS. *Bars* represent mean ± SEM for n = 6–8 rats in each group, *P < 0.05 HF vs. other groups. Bonferroni after one way ANOVA

To determine whether the changes in mSOD proteins levels correlated with the oxidative state found in the kidney, we measured TBARS in renal tissue. We found a significant increase in TBARS in the HF group, whereas this increase was prevented in the HF + WGP fed group (Fig. 6).

## Discussion

The main finding of the current study is that 16 weeks of supplementation with WGP protects against fructose-induced oxidative stress and glucose intolerance in a rat model of metabolic syndrome. The metabolic syndrome is a growing health problem and it is important to find a treatment to prevent its development. However, it is difficult to find a single treatment, because of the multiple factors that participate in this syndrome. The use of food supplements is one of the possible approaches to prevent the progression of this syndrome. The presented results show that WGP supplement prevents the evolution of the metabolic syndrome in rats.

Despite that arterial blood pressure did not change, we found that the high-fructose diet produced metabolic alterations similar to those found by others [23–26, 32]. Some studies have reported an increase in body weight [23] and arterial blood pressure [24] in rats treated with a high fructose diet, but other studies failed to find changes in these variables in Sprague–Dawley rats [25, 26]. The difference with other studies may reflect a differential response to stress of different strain rats, or the methods to measure arterial blood pressure. However, we measured arterial blood pressure in conscious rats using both radiotelemetry and the tail cuff methods. Several studies have demonstrated the ability of a high fructose diet to induce insulin resistance, reproducing the features of the metabolic syndrome in laboratory rats [23–26, 32]. Similarly to other studies, we found an increase of the area under curve of the glucose tolerance test, fasting blood glucose, plasma insulin, the HOMA index, and TBARS in plasma and renal tissue of HF-fed rats [23–26, 32].

It has been observed that high fructose fed rats develop metabolic syndrome without requiring an increased energy intake, because fructose is metabolized different than glucose ultimately producing uric acid. Uric acid is an antioxidant in the extracellular environment, but when enters the cells it induces an oxidative burst, mediated by the NADPH oxidase. This burst can induce increases in arterial blood pressure when affecting endothelial cells and in body weight. One explanation to the absence of effect on body weight and arterial pressure in our model is that in rats, uricase is expressed in high amounts so there is an efficient degradation of uric acid. Nevertheless, it has been reported that fructose induces insulin resistance even with high levels of uricase [33], and although we did not measure uricase, we did observe insulin resistance in our experimental group.

Oxidative stress is a feature of several metabolic disorders. Kunde et al. [34] evaluated the oxidative stress in the liver of mice exposed to 30 % fructose diet, and found an increase in TBARS, which did not reach significance. Similarly, Yin et al. [35] found that TBARS increased in the hippocampus and cerebral cortex in Wistar rats treated with 10 % fructose from 16 weeks. Moreover, they demonstrated that these changes were prevented by the administration of Pioglitazone, an antihyperglycemic drug. We found an increase in renal TBARS that correlates with the decrease in the levels of mSOD. The ability of WGP to prevent the increases in TBARS and the decreases in mSOD levels, suggests that WGP may be as effective as some pharmacological drugs in preventing the alterations induced by fructose.

The effects of WGP on oxidative stress and glucose intolerance found in this study could be attributed to its high content in polyphenols. These compounds are found in all plant species. They are important in plants for reproduction, growth, pigmentation and protection against pathogens [36]. Polyphenols are abundant in the mediterranean diet that is rich in fruits and wine. Grapes contain a great variety of polyphenols, including resveratrol, flavonoids, flavonols, flavones and anthocyanines. Polyphenols can exert several effects. It has been postulated that polyphenols from green tea, may reduce cholesterol and fatty acid absorption [37]. In addition, it was shown that a lyophilized grape powder reduced the overall metabolism of lipoproteins [38]. Patel et al. [39] showed that dietary polyphenols may attenuate the inflammation through modulation of the activities of NF-κB and Nrf-2.

When evaluating one particular polyphenol, it has been reported that resveratrol, extracted from grape skins, increases the activity of SIRT1 [40] and AMPK [41], and inhibits the mitochondrial ATP synthase [42]. On the other hand, EGCG has been used to inhibit several receptor tyrosine kinase activities such as the ones from vascular endothelial growth factor receptor [43] and insulin like growth factor receptor [44]. All together these results suggest that one or the combination of the different polyphenols found in WGP may produce the beneficial effects observed in the rats treated with HF and WGP on this study.

## Conclusions

In summary, the present results show that WGP supplementation prevents glucose intolerance and reduces oxidative stress in rats fed with high fructose diet. Accordingly, WGP could be used as a food supplement for the prevention of the metabolic syndrome in human. Nevertheless, further studies are needed to validate the use of this flour in humans.

## Abbreviations

WGP: wine grape powder
HF: high fructose
HOMA: homeostasis model assessment
TBARS: thiobarbituric acid reactive substances
HDL: high density lipoproteins
MS: metabolic syndrome
Nrf2: nuclear factor E2-related factor-2
mSOD: mitochondrial superoxide dismutase
GTT: oral glucose tolerance tests
AUC: area under the curve
MDA: malondialdehyde
SBTI: soy bean trypsin inhibitor
PMSF: phenylmethylsulfonyl fluoride
BSA: bovine serum albumin
PVDF: polyvinylidene difluoride

## References

[1] Haffner SM (2006). The metabolic syndrome: inflammation, diabetes mellitus, and cardiovascular disease. Am J Cardiol.

[2] Grundy SM (2008). Metabolic syndrome pandemic. Arterioscler Thromb Vasc Biol.

[3] Márquez-Sandoval F, Macedo-Ojeda G, Viramontes-Hörner D, Fernández Ballart JD, Salas Salvadó J, Vizmanos B (2011). The prevalence of metabolic syndrome in Latin America: a systematic review. Public Health Nutr..

[4] Chung SW, Kang SG, Rho JS, Kim HN, Song IS, Lee YA (2013). The association between oxidative stress and metabolic syndrome in adults. Korean J Fam Med..

[5] Yubero-Serrano EM, Delgado-Lista J, Peña-Orihuela P, Perez-Martinez P, Fuentes F, Marin C (2013). Oxidative stress is associated with the number of components of metabolic syndrome: LIPGENE study. Exp Mol Med.

[6] Lee JM, Johnson JA (2004). An important role of Nrf2-ARE pathway in the cellular defense mechanism. J Biochem Mol Biol.

[7] Lee JS, Surh YJ (2005). Nrf2 as a novel molecular target for chemoprevention. Cancer Lett.

[8] Asghar M, George L, Lokhandwala MF (2007). Exercise decreases oxidative stress and inflammation and restores renal dopamine D1 receptor function in old rats. Am J Physiol Renal Physiol..

[9] Ishii T, Itoh K, Takahashi S, Sato H, Yanagawa T, Katoh Y (2000). Transcription factor Nrf2 coordinately regulates a group of oxidative stress-inducible genes in macrophages. J Biol Chem.

[10] Kathirvel P, Chen K, Morgan K, French SW, Morgan TR (2010). Oxidative stress and regulation of anti-oxidant enzymes in cytochrome P4502E1 transgenic mouse model of non-alcoholic fatty liver. J Gastroenterol Hepatol.

[11] Kwak MK, Itoh K, Yamamoto M, Sutter TR, Kensler TW (2001). Role of transcription factor Nrf2 in the induction of hepatic phase 2 and antioxidative enzymes in vivo by the cancer chemoprotective agent, 3H-1, 2-dimethiole-3-thione. Mol Med..

[12] Zhang HF, Shi LJ, Song GY, Cai ZG, Wang C, An RJ (2013). Protective effects of matrine against progression of high-fructose diet-induced steatohepatitis by enhancing antioxidant and anti-inflammatory defences involving Nrf2 translocation. Food Chem Toxicol.

[13] Wakabayashi N, Dinkova-Kostova AT, Holtzclaw WD, Kang MI, Kobayashi A, Yamamoto M (2004). Protection against electrophile and oxidant stress by induction of the phase 2 response: fate of cysteines of the Keap1 sensor modified by inducers. Proc Natl Acad Sci USA.

[14] Ky I, Lorrain B, Kolbas N, Crozier A, Teissedre PL (2014). Wine by-products: phenolic characterization and antioxidant activity evaluation of grapes and grape pomaces from six different French grape varieties. Molecules.

[15] Choi CS, Chung HK, Choi MK, Kang MH (2010). Effects of grape pomace on the antioxidant defense system in diet-induced hypercholesterolemic rabbits. Nutr Res Pract..

[16] Lanningham-Foster L, Chen C, Chance DS, Loo G (1995). Grape extract inhibits lipid peroxidation of human low density lipoprotein. Biol Pharm Bull.

[17] Stevenson DE, Hurst RD (2007). Polyphenolic phytochemicals—just antioxidants or much more?. Cell Mol Life Sci.

[18] Roginsky AB, Ujiki MB, Ding XZ, Adrian TE (2005). On the potential use of flavonoids in the treatment and prevention of pancreatic cancer. In Vivo..

[19] Pechánová O, Rezzani R, Babál P, Bernátová I, Andriantsitohaina R (2006). Beneficial effects of provinols: cardiovascular system and kidney. Physiol Res.

[20] Manach C, Williamson G, Morand C, Scalbert A, Remesy C (2005). Bioavailability and bioefficacy of polyphenols in humans. I. Review of 97 bioavailability studies. Am J Clin Nutr.

[21] Tang SY, Halliwell B (2010). Medicinal plants and antioxidants: what do we learn from ell culture and *Caenorhabditis elegans* studies?. Biochem Biophys Res Commun..

[22] Singleton VL, Orthofer R, Lamuela-Raventos RM (1999). Analysis of total phenols and other oxidation substrates and antioxidants by means of Folin-Ciocalteu Reagent. Methods Enzymol.

[23] Choi HN, Park YH, Kim JH, Kang MJ, Jeong SM, Kim HH (2011). Renoprotective and antioxidant effects of *Saururus chinensis* Baill in rats fed a high-fructose diet. Nutr Res Pract..

[24] Dhar I, Dhar A, Wu L, Desai KM (2013). Increased methylglyoxal formation with upregulation of renin angiotensin system in fructose fed Sprague Dawley rats. PLoS One.

[25] D’Angelo G, Elmarakby AA, Pollock DM, David W, Stepp DW (2005). Fructose feeding increases insulin resistance but not blood pressure in Sprague–Dawley rats. Hypertension.

[26] Bezerra RMN, Ueno M, Silva MS, Tavares DQ, Carvalho CRO, Saad MJA, Gontijo JAR (2001). A high fructose diet induces insulin resistance but not blood pressure changes in normotensive rats. Braz J Medl Biol Res..

[27] Gutteridge JM, Halliwell B (2010). Antioxidants: molecules, medicines, and myths. Biochem Biophys Res Commun..

[28] Ou B, Hampsch-Woodill M, Prior RL (2001). Development and validation of an improved oxygen radical absorbance capacity assay using fluorescein as the fluorescent probe. J Agric Food Chem.

[29] Bors W, Saran M (1987). Radical scavenging by flavonoid antioxidants. Free Radic Res Commun..

[30] Lowry OH, Rosebrough NJ, Farr AL, Randall RJ (1951). Protein measurement with the folin phenol reagent. J Biol Chem.

[31] Ray PD, Huang BW, Tsuji Y (2012). Reactive oxygen species (ROS) homeostasis and redox regulation in cellular signaling. Cell Signal.

[32] Johnson RJ, Nakagawa T, Sanchez-Lozada LG, Shafiu M, Sundaram S, Le M (2013). Sugar, uric acid, and the etiology of diabetes and obesity. Diabetes.

[33] Tapia E, Cristóbal M, García-Arroyo FE, Soto V, Monroy-Sánchez F, Pacheco U (2013). Synergistic effect of uricase blockade plus physiological amounts of fructose-glucose on glomerular hypertension and oxidative stress in rats. Am J Physiol Renal Physiol..

[34] Kunde SS, Roede JR, Vos MB, Orr ML, Go Y-M, Park Y (2011). Hepatic oxidative stress in fructose-induced fatty liver is not caused by sulfur amino acid insufficiency. Nutrients..

[35] Yin Q-Q, Pei J-J, Xu S, Luo D-Z, Dong S-Q, Sun M-H (2013). Pioglitazone improves cognitive function via increasing insulin sensitivity and strengthening antioxidant defense system in fructose-drinking insulin resistance rats. PLoS One.

[36] Zern TL, Fernandez ML (2005). Cardioprotective effects of dietary polyphenols. J Nutr.

[37] Löest HB, Noh SK, Koo SI (2002). Green tea extract inhibits the lymphatic absorption of cholesterol and alpha-tocopherol in ovariectomized rats. J Nutr.

[38] Zern TL, West KL, Fernandez ML (2003). Grape polyphenols decrease plasma triglycerides and cholesterol accumulation in the aorta of ovariectomized guinea pigs. J Nutr.

[39] Patel R, Maru G (2008). Polymeric black tea polyphenols induce phase II enzymes via Nrf2 in mouse liver and lungs. Free Radic Biol Med.

[40] Howitz KT, Bitterman KJ, Cohen HY, Lamming DW, Lavu S, Wood JG (2003). Small molecule activators of sirtuins extend *Saccharomyces cerevisiae* lifespan. Nature.

[41] Zhang F, Sun C, Wu J, He C, Ge X, Huang W (2008). Combretastatin a-4 activates AMP-activated protein kinase and improves glucose metabolism in db/db mice. Pharmacol Res.

[42] Gledhill JR, Montgomery MG, Leslie AG, Walker JE (2007). Mechanism of inhibition of bovine F1-ATPase by resveratrol and related polyphenols. Proc Natl Acad Sci USA.

[43] Kondo T, Ohta T, Igura K, He C, Ge X, Huang W (2002). Tea catechins inhibit angiogenesis in vitro, measured by human endothelial cell growth, migration and tube formation, through inhibition of VEGF receptor binding. Cancer Lett.

[44] Shimizu M, Deguchi A, Hara Y, Moriwaki H, Weinstein IB (2005). EGCG inhibits activation of the insulin-like growth factor-1 receptor in human colon cancer cells. Biochem Biophys Res Commun..

